# Impact of Infection Dose and Previous Serum Antibodies against the Locus of Enterocyte Effacement Proteins on *Escherichia coli* O157:H7 Shedding in Calves following Experimental Infection

**DOI:** 10.1155/2015/290679

**Published:** 2015-06-08

**Authors:** L. Martorelli, C. J. Hovde, D. A. Vilte, A. Albanese, E. Zotta, C. Ibarra, R. J. C. Cantet, E. C. Mercado, A. Cataldi

**Affiliations:** ^1^Instituto de Patobiología, CICVyA, INTA, 1686 Hurlingham, Argentina; ^2^School of Food Science, University of Idaho, Moscow, ID 83844-3025, USA; ^3^Laboratorio de Fisiopatogenia, Departamento de Fisiología, Facultad de Medicina, Universidad de Buenos Aires, 1121 Ciudad Autónoma de Buenos Aires, Argentina; ^4^Facultad de Agronomía, Universidad de Buenos Aires-CONICET, 1417 Ciudad Autónoma de Buenos Aires, Argentina; ^5^Instituto de Biotecnología, CICVyA, INTA, 1686 Hurlingham, Argentina

## Abstract

*Escherichia coli* O157:H7 is the main causative agent of haemolytic uremic syndrome. Cattle are the main reservoir of these bacteria, and have been shown to develop immune response to colonization. Our aim was to investigate the faecal shedding pattern of *E. coli* O157:H7 in calves challenged intragastrically with either 10^8^ or 10^10^ CFU, as well as the ability of specific preexisting antibodies to reduce shedding of the pathogen. Shedding was analysed by direct counting as well as enrichment of rectoanal mucosal swabs. Statistical analysis was performed using a linear model for repeated measures with and without the inclusion of preexisting antibodies against the carboxy-terminal fraction of intimin-*γ* (*γ*-intimin C280) as a covariable. Results suggest that there is a statistical difference in the area under the shedding curves between both doses for 14 as well as 28 days after challenge (*p* = 0.0069 and 0.0209, resp.). This difference is increased when the prechallenge antibodies are taken into account (*p* = 0.0056 and 0.0185). We concluded that the bacterial dose influences shedding on calves experimentally challenged and that preexisting antibodies against *E. coli* O157:H7 *γ*-intimin C280 could partially reduce faecal excretion.

## 1. Introduction

Enterohaemorrhagic* Escherichia coli* (EHEC) O157:H7 is a major etiologic agent of diseases in humans, whose clinical spectrum includes diarrhoea, haemorrhagic colitis, and haemolytic uremic syndrome (HUS), the leading cause of chronic renal failure in children in Argentina and several other countries [1, 2].

Cattle are the main reservoir of EHEC O157:H7, which predominately colonizes the lymphoid follicle-dense mucosa at the terminal rectum and the rectoanal junction (RAJ) [3]. Faecal contamination of meat during slaughter, the use of raw faeces as fertilizers, and the contamination of drinking water are the major ways by which this microorganism can enter the human food chain [4–6].

This bacterium produces Shiga toxins types 1 and/or 2 [7–9], which are responsible for systemic damage, particularly of the vascular endothelium of kidneys and brain, with severe renal and neurological sequelae in children and the elderly. In addition to Shiga toxins,* E. coli* O157:H7 is characterized by other virulence-associated traits which enables it to colonize the intestinal mucosa of humans and animals with a characteristic histopathological lesion known as “attaching and effacing” (A/E). A large chromosomal pathogenicity island called Locus of Enterocyte Effacement (LEE) is associated with A/E activity [10–12]. The LEE encodes a type three secretion system (TTSS) that translocates effector proteins responsible for the A/E lesion into the host cell. Intimin, a bacterial outer membrane protein, binds to Tir, the translocated intimin receptor in the host cell membrane, and this binding leads to the formation of the A/E lesion. Tir, EspB, and other LEE-encoded proteins are translocated into the host cell through a transiently produced filamentous structure [13], which consists of an assembly of EspA subunits [14] and contributes, in turn, to the creation of a pore in the eukaryotic cell membrane.

Ample evidence suggests that infected animals can excrete the bacteria in their faeces from a few days to months [6, 15]. Key features associated with the rate of colonization are the age of the animals and genetic markers in the pathogen.

Many virulence factors of* E. coli* O157:H7 induce an immune response during the course of natural as well as experimental infections in cattle. Oral inoculation of calves and steers with* E. coli* O157:H7 promotes an increase in serum antibody titres against the O157 lipopolysaccharide and neutralizing antibodies to Shiga toxins. Recently, Bretschneider et al. [16] demonstrated that cattle respond serologically to Intimin and EspB of* E. coli* O157:H7 during the course of experimental infection. Furthermore, several authors have reported that calves and cattle shed fewer bacteria after several experimental inoculations, which could be related to a partially protective immune response elicited by the pathogen [17–19].

Vaccination is one of the preslaughter interventions that shows promise to reduce EHEC O157:H7 occurrence in cattle. We, along with other groups, have demonstrated that vaccination of calves with type three secretion injection apparatus proteins results in reduced excretion of EHEC O157:H7 after experimental infection in cattle with an oral challenge dose of 10^10^ CFU [20–23].

We therefore assessed if the level of colonization depends on the bacterial challenge dose and whether previous antibodies against the carboxy-terminal fraction of *γ*-intimin (*γ*-intimin C280) could reduce faecal excretion of the pathogen. Furthermore we assessed the humoral immune response elicited in response to the challenge. For this purpose, we compared the effect of two different doses of oral inoculation in calves regarding the colonization status and the associated serum immune response with shedding.

## 2. Materials and Methods

### 2.1. Animals

Nine four-month-old conventionally reared Holando-Argentino male calves were obtained from a dairy farm in Buenos Aires Province, Argentina, and housed in biosafety level 2 containments rooms at the Instituto Nacional de Tecnología Agropecuaria (INTA) Experimental Station. All calves were acclimatized to the containments room for 1 week prior to the challenge and were deemed healthy by clinical examination and treated prophylactically upon arrival with 1% ivermectin to control intestinal nematodes. Calves were fed alfalfa pellets, with free access to hay and water.

Prior to the challenge, calves were confirmed twice (one month and three days before inoculation) to be negative for* E. coli* O157:H7 by enrichment of rectoanal mucosal swabs followed by immunomagnetic separation, and serum specific antibodies were also assessed as described below. All animal experiments were performed with the approval of the INTA Animal Welfare Committee.

### 2.2. Strain

The challenge strain of* E. coli* O157:H7 selected for the experimental infection was 438/99. This strain was isolated from a healthy cow and was used previously in experimental studies [22]. It was selected for spontaneous resistance to nalidixic acid to facilitate recovery from rectoanal mucosal swabs and tissues and possesses the genes for enterohemolysin, *γ*-intimin, EspA, EspB, Stx2, and the pO157 plasmid.

### 2.3. Experimental Infection

Animals were randomly assigned to Groups 1 (*n* = 5) and 2 (*n* = 4) and challenged intragastrically with the bacterium. Group 1 received 10^8^ CFU, whereas Group 2 was challenged with a dose of 10^10^ CFU of* E. coli* O157:H7 strain 438/99 in 15 mL of sterile PBS. Randomization was performed to assure that both groups were composed of animals with similar weight distribution (approximately 100 and 120 kg, resp.).

### 2.4. Sample Collection

Rectoanal mucosal swabs were taken at 0, 2, 5, 8, 11, 14, 17, 20, 24, and 28 days after the challenge [24]. In addition, serum samples were taken at 0, 8, 17, and 28 days after the oral bacterial challenge. Necropsy was performed at day 28 after challenge and tissue sections from the ileum, cecum, and rectoanal junction were obtained and evaluated for the presence of bacteria and mucosal antibodies and for histopathology.

One animal from Group 2 was euthanized 20 days after the challenge for health issues according to the INTA Animal Welfare Committee standards.

### 2.5. *E. coli* O157:H7 Shedding

The magnitude and duration of the fecal excretion of viable* E. coli* O157:H7 were followed by culture of rectoanal mucosal swabs, as previously described [25]. Briefly, bacterial CFU/swab was determined by vortexing the swabs in Trypticase soy broth (TSB, Oxoid, Basingstoke, UK), plating serial dilutions on Sorbitol MacConkey agar (Oxoid, Basingstoke, UK) containing 20 *μ*g/mL nalidixic acid (Sigma, St. Louis, USA), 2.5 *μ*g/mL potassium tellurite, and 0.05 *µ*g/mL cefixime (CT-SMAC). When direct cultures were negative, swabs were enriched at 37°C for 18 h and 1 mL of this culture was subjected to* E. coli* O157 immunomagnetic separation (IMS) performed according to the manufacturer's instructions (Dynabeads anti-*E. coli* O157, Invitrogen Dynal AS, Oslo, Norway). The bead-bacterium mixture was plated on CT-SMAC. Samples that resulted culture-positive by IMS were considered positive (10 CFU), while samples culture-negative by IMS were deemed negative. Non-sorbitol-fermenting colonies were tested for* E. coli* O157 LPS by latex agglutination (Oxoid, Basingstoke, UK) and confirmed by a multiplex PCR for the stx1, stx2, eae, and rfbO157 genes [26–28]. Tissues obtained at necropsy were cultured to detect* E. coli* O157:H7 after IMS as described above.

### 2.6. Production of Recombinant* E. coli* O157:H7 Proteins

EspB and *γ*-intimin C280 genes from the* E. coli* O157:H7 strain 146N were cloned in pRSET-A vector (Invitrogen Corp., Carlsbad, CA) and expressed in* E. coli* BL21 (D3)/pLysS, as described previously [29]. The amino-terminal-His-tagged proteins were purified from the lysates by affinity chromatography on nickel-agarose columns (ProBond nickel-chelating resin; Invitrogen Corp.), eluted under denaturing conditions, and dialyzed in PBS at pH 7.4.

### 2.7. Antibody Response

All sera samples taken before and during the experiment were analyzed by ELISA to detect specific antibodies against* E. coli* proteins EspB and *γ*280-intimin as described elsewhere [29]. Briefly, 96-well Nunc-Immuno MaxiSorp assay plates (Nunc, Roskilde, Denmark) were coated overnight at 4°C with 100 *µ*L of either *γ*280-intimin or EspB at 1 *µ*g/mL in carbonate buffer pH 9.6. After three washes with PBS pH 7.4 containing 0.05% Tween 20 (PBS-T), nonspecific binding sites were blocked with 3% skimmed milk in PBS for 1 h at 37°C. After that, washes were repeated, and sera diluted 1/50 in PBS-T were added (100 *µ*L/well). Plates were incubated for 2 h at 37°C. For each plate, two wells were incubated with PBS-T alone (negative control), and a known positive sample was included. Each sample was analyzed in duplicate. After washing with PBS-T, wells were incubated for another hour with 100 *µ*L of sheep anti-bovine IgG, IgG1, IgG2, or IgA conjugated with horseradish peroxidase (Bethyl Laboratories, Montgomery, USA), at dilutions of 1 : 10000 in PBS-T. Plates were washed three times with PBS-T. Finally, ABTS [2,2-azino-di (3-ethyl-benzthiazoline sulphonic acid)] (Amresco, Solon, USA) in citrate-phosphate buffer pH 4.2 plus 0.01% H_2_O_2_ (100 *µ*L/well) was added. Reactions were stopped after 10 min with 100 *µ*L/well of 5% SDS and read at 405 nm (OD_405_) in a BioTek ELx808 microplate reader (BioTek Instruments, Winooski, USA). Mucosal antibodies from intestinal tissue samples taken at necropsy were obtained as described elsewhere [30] and assessed by ELISA for the presence of specific antibodies as described above. The specificity of antibodies was confirmed by Western blot.

### 2.8. Histopathological Examination

One segment of ileum, cecum, and rectoanal junction (approximately 20 g each) from each animal was obtained at necropsy and immediately fixed in neutral buffered 10% formalin, dehydrated with alcohol, and embedded in paraffin. Sagittal cuts (5 *µ*m) were made with a microtome (Leica RM 2125, Wetzlar, Germany), stained with hematoxylin and eosin (H&E), and mounted on 2% silane-coated slides. Tissue sections were observed by light microscopy (Nikon Eclipse 200, NY, USA).

### 2.9. Immunohistochemistry

Immunohistochemistry was performed on tissue segments that were positive for* E. coli* O157:H7 culture, as described above. Briefly, dewaxed sections were blocked for endogenous peroxidase with H_2_O_2_ 3% in methanol for 10 min. Later, the slides were preincubated with no immune rabbit serum at room temperature for 30 min. Then, the primary rabbit anti-O157 LPS antibody, which was kindly provided by Susana Bruno (Antisera Service (ANLIS, Argentina)), was added (1 : 100). The sections were incubated with the primary antibody in a humidity chamber at 4°C overnight. The immunoperoxidase technique was then performed following the protocol from the RTU Vectastain Kit (Vector, Peterborough, UK). The antigen was revealed by diaminobenzidine (DAB, Vector). Finally, the sections were counterstained by Meyer's Hematoxylin and mounted for observation. Specificity tests were performed by omitting the primary antiserum in the staining.

### 2.10. Statistical Analysis

Data analyzed were bacterial shedding of calves and levels of antibodies. To attain normality, both set of variables were transformed using procedures described by Box and Cox [31]. The transformation parameter was obtained after a grid search in the open interval (−0.95, 0.95). Resulting variables were analyzed using linear models for repeated measures [32], as in Vilte et al. [22, 23].

For the transformed CFU (TCFU) variable describing the shedding of calves effects in the model were dose of* E. coli* O157:H7 (as a classification variable with two levels: 10^8^ and 10^10^ CFU), the covariate initial level of antibodies nested within dose (i.e., one covariate per dose), and a linear term for the covariate day of measure interacting with dose, so as to account for the daily trend within treatment. The dependence structure among repeated measures within animal within dose was modeled with a Gaussian covariance matrix. The area under the curve (AUC) was obtained using estimable linear functions [33]. The hypothesis of interest was whether the estimated AUC from the two doses differ at different times (14 and 28 days), and including or excluding the effect of the initial antibody level. All tests of hypotheses had their degrees of freedom corrected by the procedure of Kenward and Roger [34], and *p* values smaller than 0.05 were considered to be significant.

Differences in antibodies were tested from either estimable contrasts or differences in least-square means [33]. Models used included different effects according to antibody. In all cases, the residual covariance structure was 4-banded Toeplitz for the four different measures, within animal. All analyses were performed using Proc Mixed of SAS version 9.2 (SAS, 2013).

## 3. Results

### 3.1. *E. coli* O157:H7 Shedding after Challenge

**Figure 1:**
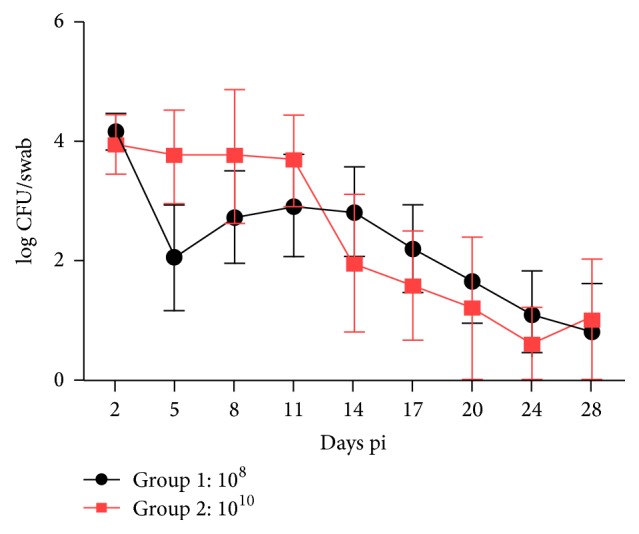
Shedding pattern of calves inoculated with either 10^8^ or 10^10^ CFU of* E. coli* O157:H7. Data are shown as log CFU/swab ± S.E.M.

In order to observe the effect of challenge dose on total shedding and kinetics of* E. coli* O157:H7, two groups of calves were orally challenged either with 10^8^ or with 10^10^ CFU of* E. coli* O157:H7. The kinetics of fecal shedding in both groups is shown in Figure 1. To understand if there is a relationship between dose and fecal shedding, we performed a statistical analysis using a linear model for repeated measures (SAS) analysis (Table 1). All main effects and covariates displayed a highly significant effect on TCFU: dose 10^10^ produced more TCFU than 10^8^. For the covariable anti-*γ*-intimin C280 antibody (dose), the higher the level of antibodies the smaller the number of TCFU. Finally, the linear covariate dose by day showed smaller levels as the days passed. Cubic and quadratic responses (not shown in Table 1) were far from being significant. When anti-EspB antibodies were used as covariable, no significant effect of the TCFU was observed. The results of the tests of hypothesis of differences in AUC by dose are displayed in Table 2. All tests produced significant differences in AUC due to dose and day (14 or 28).

There was a significant difference in shedding between the groups. The calves that received 10^8^ CFU shed fewer bacteria for 14 days as well as 28 days, compared to the ones that were challenged with 10^10^ CFU. When the levels of preexisting IgG antibodies against *γ*-intimin C280 were considered in the model, the difference became even more significant (see *p* values in Table 2).

One animal from each group was excreting* E. coli* O157:H7 on day 28 after the challenge, and the challenge strain was isolated from rectoanal junction tissue obtained from these animals at necropsy. The bacterium was also detected by immunohistochemistry (see below).

### 3.2. Humoral Immune Response to* E. coli* O157:H7 Challenge

**Figure 2:**
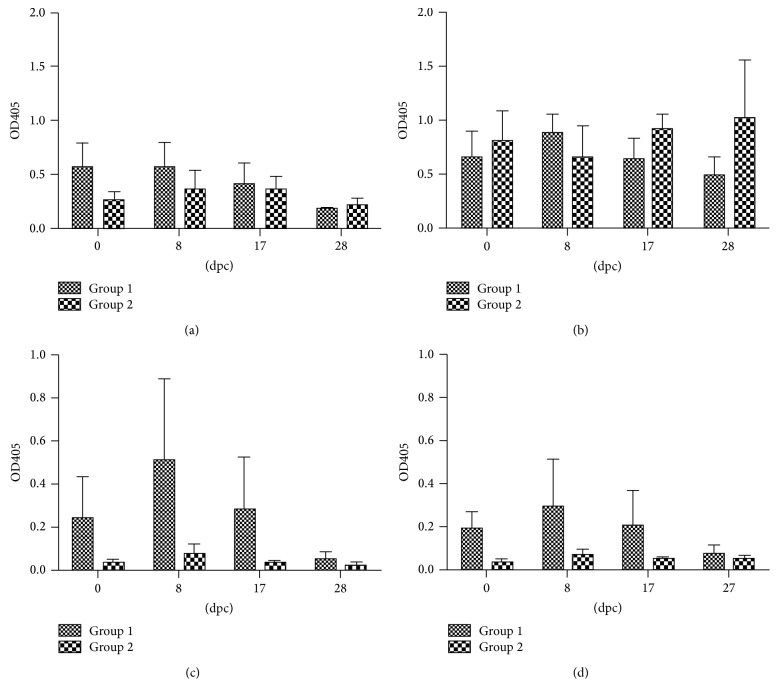
IgG ((a), (b)) and IgA ((c), (d)) antibodies against *γ*-intimin C280 ((a), (c)) and EspB ((b), (d)) in calves challenged with 10^8^ or 10^10^ CFU of* E. coli* O157:H7. Data are shown as mean OD405 ± S.E.M.

Serum IgG and IgA antibodies were assessed by ELISA on the day of* E. coli* O157:H7 challenge (0 dpc) and at different time points after the challenge. Results are shown in Figure 2. As expected, specific IgG was higher than IgA for both antigens tested. Furthermore, most of the IgG was IgG1, whereas IgG2 was very low (data not shown). Higher OD_405_ values were obtained for IgG antibodies against EspB than against *γ*-intimin C280, and these levels were very similar for both groups.

IgA response against any of the antigens was not detected in the group that received 10^10^ CFU whereas the level of IgA specific for both antigens rose in the calves challenged with the lowest dose reaching, at day 8 after the challenge, levels similar to those of IgG. In spite of this, the difference was not statistically significant.

No neutralizing activity against Stx2 was observed (data not shown).

### 3.3. Mucosal Immune Response to* E. coli* O157:H7 Challenge

**Figure 3:**
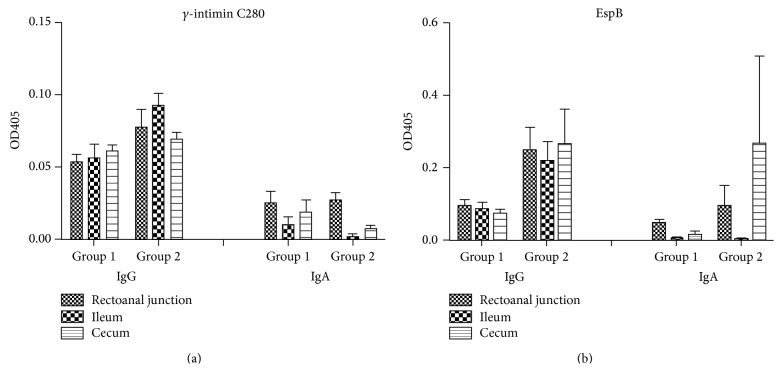
Mucosal IgG and IgA antibodies against EspB and *γ*-intimin C280 in calves challenged with 10^8^ or 10^10^ CFU of* E. coli* O157:H7. Data are shown as mean OD405 ± S.E.M.

Specific mucosal IgG and IgA antibodies were assessed in tissue segments of rectoanal junction, ileum, and cecum. Results are shown in Figure 3.

IgG against both EspB and *γ*-intimin C280 was found significantly higher than IgA in both groups in the three segments assessed (*p* < 0.0001).

IgG and IgA antibodies against EspB were significantly higher in RAJ and cecum of Group 2 than in Group 1, whereas no significant differences were found in antibodies against *γ*-intimin C280 between groups.

### 3.4. Histopathology

Upon histopathological examination, ileum and cecum from Groups 1 and 2 presented histological signs of inflammation and mild epithelial necrosis with detachment of the surface epithelium (Figure 4 Supplementary Material, in Supplementary Material available online at http://dx.doi.org/10.1155/2015/290679). Taking into account that the preferential attachment site of the bacteria is the rectoanal junction, it is important to mention that a normal histoarchitecture with polymorphonuclear leukocytes infiltrations was observed in this tissue (Figure 4 Supplementary Material).

### 3.5. Immunohistochemistry

*E. coli* O157:H7 was detected by using an antibody against O157 in segments of rectoanal junction of two calves that continued to excrete at day 28 after inoculation (Figure 5 Supplementary Material).

## 4. Discussion

During this study, two groups of randomly assigned calves were intragastrically challenged with either 10^8^ or 10^10^ CFU of* E. coli* O157:H7. We observed a statistically significant difference in the area under the shedding curves between the two doses of bacterial challenge used in this study for both 14 and 28 days, the group that received the lower dose being the one that shed less bacteria, suggesting a relationship between the challenge dose and the bovine intestinal colonization.

Furthermore, when the preexisting antibodies against *γ*-intimin C280 were included in the model, this difference became even more significant. This finding suggests that previous antibodies against this protein could partially reduce* E. coli* O157:H7 carriage in calves.

Intimin has been shown to be a target of humoral immune responses in different host species and animal models such as humans [35–37], cattle [16, 20–23, 29], rabbits [24], pigs [38], and mice [39, 40]. These antibodies have been proved to be effective in avoiding epithelial colonization by* E. coli* O157:H7 [41] and can be obtained through vaccination [20–23] as well as experimental infections [22, 23]. However, this response is not completely protective since animals continue to excrete the bacteria despite a reduction in shedding and can be reinfected. In the present conditions, we cannot differentiate if the preexisting antibodies against *γ*-intimin C280 came from passive immunization through colostrum from their mothers after birth or from the fact that animals were infected by EHEC in the field.

During this study, we did not observe a statistically significant increase in serum antibodies after challenge against any of the antigens tested. However, there was a difference between IgG and IgA antibodies, which could be explained considering that IgG concentration in bovine serum is higher than IgA and has a longer half-life [42, 43].

Interestingly, no IgA response was detected against either EspB or *γ*-intimin C280 in the serum of calves from Group 2. Naylor et al. [17] have demonstrated that young calves develop serum IgG and IgA antibody responses to O157 and H7, and the IgA but not IgG titers decreased after colonization following a repeated challenge. Furthermore, it was found [16] that the IgA titers decreased only to factors having a known (Eae and Tir) or highly probable function in colonization (EspB), whereas those to O157 increased. These results suggest that serum IgA antibodies against virulence factors involved in colonization may be consumed in response to challenge.

A mild mucosal immune response was observed in both groups in response to* E. coli* O157 challenge, with higher IgG than IgA antibody levels, as expected, since this immunoglobulin predominates in the mucosa of ruminants [44]. Significantly higher levels of IgG and IgA antibodies against EspB were found in RAJ and cecum of the calves that received the higher dose of bacteria. Interestingly, these animals had very low levels of IgA antibodies in serum. These results suggest that the challenge dose influences the colonization rate and the immune response elicited, at least, in some mucosal epitheliums. Nart et al. [30] have shown that the* E. coli* O157:H7 colonization induces a local mucosal antibody response, which is directed at several membrane-associated and secreted proteins. Similarly, adult cattle develop colonic and rectal mucosal antibodies of the IgG and IgA classes to* E. coli* O157:H7 antigens following experimental infection; however, these titers are low [16]. This could be explained by the evidence provided by Hoffman et al. [45] who suggested that Stx2 produced by* E. coli* O157:H7 during the course of infection may reduce lymphoproliferative responses in intraepithelial as well as peripheric lymphocytes. By contrast, the strain used in our study was Stx2+. Therefore, its immunosuppressive effect must be taken into account since it could explain the low mucosal antibody response that was observed. It would be interesting to further study this response in other mucosal sites such as saliva or nasal swabs.

This study has led us to conclude that the dose of experimental challenge with* E. coli* O157:H7 influences the colonization rate on the bovine intestinal tract and that preexisting antibodies against *γ*280-intimin may have a protective role against colonization. We have also concluded that oral experimental challenge with a Stx2+ strain induces a mild mucosal immune response.

This finding suggests a relationship between colonization, shedding, and immunity. Although further studies are required to elucidate the role of antibodies and cellular immunity in the excretion dynamics of* E. coli* O157:H7, the enhancement of immune responses can be proposed as a potential prophylactic measure to control bovine carriage of* E. coli* O157:H7.

## Supplementary Material

Figure 4: Histopathology of ileum, cecum and RAJ from animals of Group 1 and Group 2 stained with hematoxylin and eosin.
Figure 5: Histopathological immunodetection of E. coli O157:H7 in intestine.

## Figures and Tables

**Table 1:** Tests of the effects into the model for transformed CFU.

Effect	Numerator df	Denominator df	*F*-value	Pr < *F*
Dose	2	36.4	57.63	<0.0001

Anti-*γ*-intimin C280 antibody (dose)	2	33.5	8.58	0.0010

Anti-EspB antibody (dose)	2	35.4	0.72	0.4944

Day × dose	2	40.4	16.77	<0.0001

**Table 2:** Test of hypotheses on differences of areas under the curve for different doses and at different days.

Null hypothesis	Covariate level of antibody considered	Day	Result of the estimate	*p* value
AUC 1 = AUC 2	No	14	AUC 1 < AUC 2	0.0069
AUC 1 = AUC 2	Yes	14	AUC 1 < AUC 2	0.0056
AUC 1 = AUC 2	No	28	AUC 1 < AUC 2	0.0209
AUC 1 = AUC 2	Yes	28	AUC 1 < AUC 2	0.0185
